# Fingersomatotopy in area 3b: an fMRI-study

**DOI:** 10.1186/1471-2202-5-28

**Published:** 2004-08-20

**Authors:** Danielle van Westen, Peter Fransson, Johan Olsrud, Birgitta Rosén, Göran Lundborg, Elna-Marie Larsson

**Affiliations:** 1 Dept of Diagnostic Radiology, Lund University Hospital, 221 85 Lund, Sweden; 2 Cognitive Neurophysiology, MR Research Centre, N8, Dept of Clinical Neuroscience, Karolinska University Hospital, 171 76 Stockholm, Sweden; 3 Dept of Hand Surgery, University Hospital, 205 02 Malmö, Sweden

## Abstract

**Background:**

The primary sensory cortex (S1) in the postcentral gyrus is comprised of four areas that each contain a body map, where the representation of the hand is located with the thumb most laterally, anteriorly and inferiorly and the little finger most medially, posteriorly and superiorly. Previous studies on somatotopy using functional MRI have either used low field strength, have included a small number of subjects or failed to attribute activations to any area within S1. In the present study we included twenty subjects, who were investigated at 3 Tesla (T). We focused specifically on Brodmann area 3b, which neurons have discrete receptive fields with a potentially more clearcut somatotopic organisation. The spatial distribution for all fingers' peak activation was determined and group as well as individual analysis was performed.

**Results:**

Activation maps from 18 subjects were of adequate quality; in 17 subjects activations were present for all fingers and these data were further analysed. In the group analysis the thumb was located most laterally, anteriorly and inferiorly with the other fingers sequentially positioned more medially, posteriorly and superiorly. At the individual level this somatotopic relationship was present for the thumb and little finger, with a higher variability for the fingers in between. The Euclidian distance between the first and fifth finger was 17.2 mm, between the first and second finger 10.6 mm and between the remaining fingers on average 6.3 mm.

**Conclusion:**

Results from the group analysis, that is both the location of the fingers and the Euclidian distances, are well comparable to results from previous studies using a wide range of modalities. On the subject level the spatial localisation of the fingers showed a less stringent somatotopic order so that the location of a finger in a single subject cannot be predicted from the group result.

## Background

The first somatotopic maps of the homuncular organisation of the primary somatosensory cortex (S1) were established in 1937 by using intra-operative electrical stimulation of the brain surface [1]. Subsequent, non-invasive investigations in humans on the hand representation in S1 have described a somatotopic organisation along the central sulcus with the thumb located laterally, anteriorly and inferiorly to the little finger [2-6]. Studies in non-human primates have revealed the cytoarchitectonic subdivisions of S1, namely areas 3a, 3b, 1 and 2, that outline the cortex in the postcentral gyrus [7]. Area 3a occupies the fundus of the central sulcus, area 3b the anterior wall of the postcentral gyrus, area1 its crown and area 2 its posterior wall. Each area contains a fairly complete map of the body surface and is the cortical representation of different somatosensory receptors. In area 3b the neurons are predominantly responsive to stimulation of cutaneous receptors. As opposed to neurons in area 1, that also receive input from cutaneous receptors, those in area 3b possess discrete receptive fields with a homuncular organisation that may be more distinct [8].

Previous studies on somatotopy in the hand area using functional Magnetic Resonance Imaging (fMRI) have yielded varying results. Gelnar et al. failed to show a somatotopy in S1 when applying vibratory stimuli to three of the fingers of the right hand [5]. Maldjian et al. demonstrated somatotopy in 3 out of 5 subjects [3]. Similarly, Kurth et al., using electrical stimulation of two fingers, found somatotopically arranged activation patterns in 5 out of 20 subjects [9]. In a follow-up study where activation of all fingers in area 3b was found in 7 out of 10 subjects, the same authors reported a general somatotopy, without further specification [4].

Methods differ considerably between these three studies with regard to anatomical considerations, number of subjects studied, and the field strength used. Maldjian et al. used the highest field strength, 4 Tesla (T), while both Kurth et al. and Gelnar et al. used 1.5 T [3-5]. However, Maldjian et al. did not contribute activations to any area in S1. Also Maldjian et al. included the smallest number of subjects (5); data from one were discarded due to motion artefacts and group analysis was based on data from the remaining 4.

In the present study we readdressed the issue of somatotopy in the hand area as assessed with fMRI. Our aim was to optimise results by including a larger number of subjects, by focussing on area 3 b, where homuncular organisation expectedly is most distinct and by performing fMRI at 3 Tesla (T).

## Results

Tactile stimulation of the fingers of the dominant hand yielded activation in contra-and ipsilateral S1, contra- or bilateral secondary sensory cortex (S2), ipsilateral cerebellum, and in some subjects the contralateral thalamus. Significant activation for all five fingers in area 3b was present in seventeen subjects and these data were further analyzed. In the one volunteer excluded from the analysis, activation was present for four fingers. The spatial distribution of the activations in the contralateral S1 for tactile stimulation versus rest in one subject is shown in Figure 2.

A somatotopic organisation with the representation of the thumb located laterally to the little finger was present in 16 out of 17 subjects, with the thumb located anteriorly to the little finger in 14 out of 17 subjects and with the thumb located inferiorly to that of the little finger in 16 out of 17 subjects.

Group averages of the distances from D2 to D1 (D2-D1), D3 to D1(D3-D1), D4 to D1 (D4-D1) and D5 to D1 (D5-D1) are presented in the Table and shown as graphs in Figure 3. Combined these indicate a strict somatotopy with the distance to D1 increasing for every finger in each of the three directions. Distances to D1 were compared for neighbouring fingers. In the medial-lateral direction, the distance D4-D1 was different from D3-D1. In the anterior-posterior direction a significant difference was observed between D4-D1 and D3-D1. Finally, in the superior-inferior direction the distances D2-D1 and D3-D1 as well as D3-D1 and D4-D1 differed; the location of D2, as determined by its distance to D1, was different from the location of D1 [0, 0, 0]; no difference was found between the distances D5-D1 and D4-D1. Considering that the three coordinates [x, y and z] together define one point in the 3D Cartesian space, the coordinates of D3, D4 and D5, differed from those of D1, p now <0.05/3, corrected for multiple comparisons (not in Table).

The Euclidian distance from D1 to D2 was 10.6 mm (SEM ± 1.5). The distance from D2 to D3 was 5.5 mm (± 0.9), from D3 to D4 7.4 mm (± 1.1) and from D4 to D5 6.8 mm (± 1.2), resulting in an average for D2-D3, D3-D4 and D4-D5 of 6.6 mm.

The spatial extension of the representation of the hand in area 3b, defined as the Euclidian distance between D1 and D5 was 17.2 mm (± 2.0 mm).

## Discussion

In the group average from the present study the strict somatotopic organisation in the primary sensory cortex known from studies using a variety of modalities, was reproduced [2-6]. The fingers' average activations were laid out on the body map, with the thumb located most laterally, anteriorly and inferiorly and the little finger most medially, posteriorly and superiorly and the remaining fingers in between, the distance to the thumb increasing for every finger in each of the three directions. In individual subjects the arrangement in the hand representation with the thumb located laterally, anteriorly and inferiorly to the little finger is frequently found, while the remaining fingers may or may not display the orderly lateral-to-medial, anterior-to-posterior and inferior-to-superior organisation 'D1-D2-D3-D4-D5' [2,3,10,11]. We chose to present group averages as it is our belief our results would have greater significance if a regular somatotopy was present at the group level. Body maps in non-human primates demonstrating the regular sequence mentioned above, were established with cortical single unit recordings and are supposedly the golden standard. The present study is based on data from 20 subjects, generating results available for analysis from 18 of these; the spatial representation of all fingers in area 3b of the primary somatosensory cortex was localised in 17 subjects.

The average extension of the hand representation in area 3b of 17 mm with a somatotopic arrangement of fingers 1-5 as described above is consistent with results from previous studies using a range of modalities [2-4,7]. Also the mean distance between D2-D3, D3-D4 and D4-D5 of 6.6 mm and 6.3 mm for main and differential effects, respectively, is in good agreement with human electrophysiological and fMRI data [2,4]. The larger distance between the thumb and index finger as compared to distances between subsequent fingers suggests a larger representation for the thumb. This finding is in agreement with results from a study using electrocorticography with subdural electrodes in three patients [1,12].

With the spatial resolution used in this study, 3 × 3 × 3 mm^3^, resampled to 1.5 × 1.5 × 1.5 mm^3^, activation for all five fingers was found in 17 of 18 subjects. For comparison, Kurth et al. used a resolution of 1.7 × 1.7 × 1.7 mm3 and electrical stimulation with ring electrodes and found activation in area 3b for all five fingers in 7 out of 10 subjects (70%) [4]. The present study showed activations for all fingers in 94 % of subjects. This difference might be due to the higher field strength used in this study as the higher magnetisation vector and sensitivity to changes in susceptibility increase the signal-to-noise ratio.

In one subject excluded from the analysis, activation was present for four fingers and another was excluded due to general lack of activation. Lack of activation may be due to subjects being 'low-activators' in fMRI experiments, due to inadequate stimulation or to some other, unknown factor.

The type of stimulation used is decisive of what area in S1 can be expected to be activated. For example, neurons in area 3a are responsive to deep receptor and proprioceptive stimulation and in one study punctate tactile stimulation did not activate area 3a [13]. Also, receptive fields are maximally focused in area 3b, while in area 1 receptive fields become larger and more complex. In area 2, receptive fields are even more complex with reduplications. This combined knowledge made area 3b the area of our choice to study somatotopy and explains why we chose not to report on activations in areas 3a, 1 and 2.

**Figure 1:**
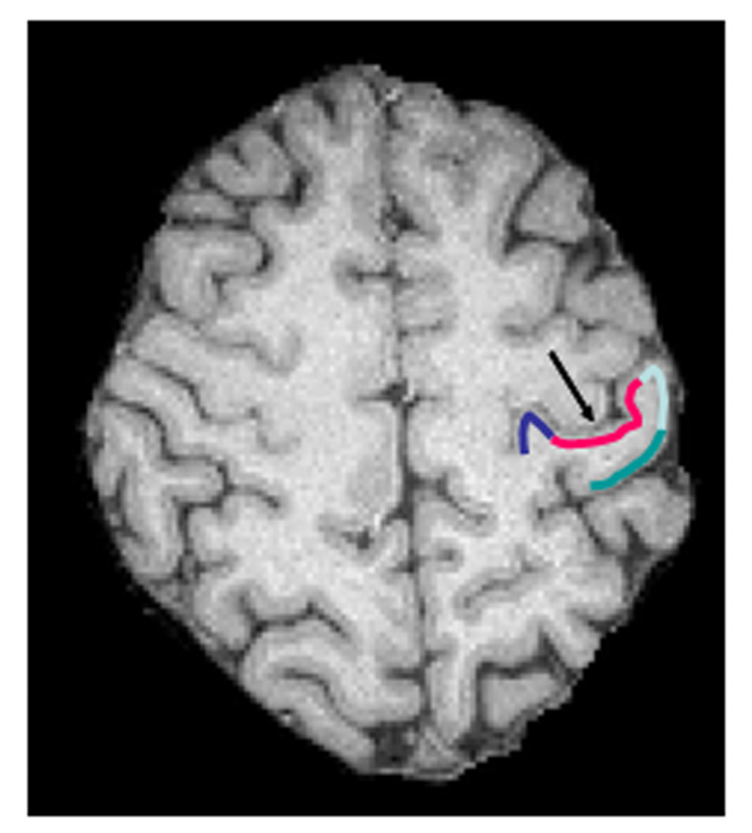
Areas of S1 as defined in cytoarchitectonic studies on 10 post-mortem brains [18, 19]: area 3a occupies the fundus of the central sulcus (dark blue), area 3b the anterior wall of the postcentral gyrus (red), area1 its crown (light blue) and area 2 its posterior wall (green). The black arrow indicates the central sulcus.

**Figure 2:**
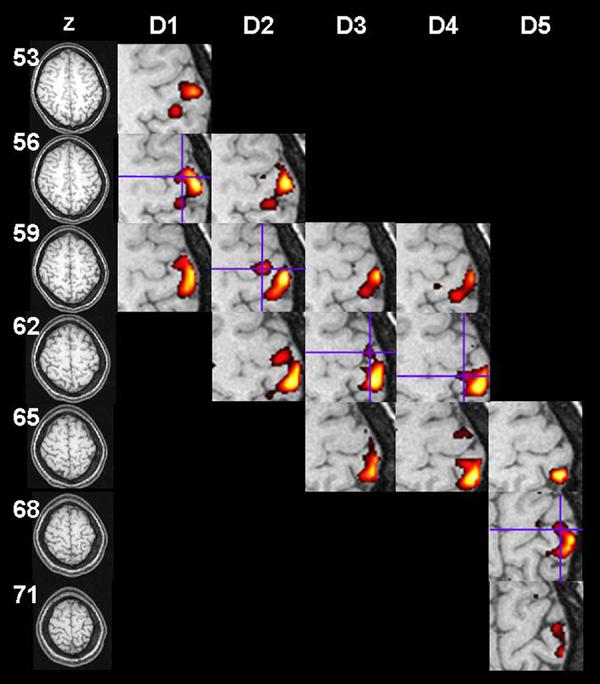
Activation in the contralateral somatosensory cortex during tactile stimulation of the fingers of the right hand versus rest in a single subject. The first column shows transverse anatomical image with z-coordinate indicated. Subsequent columns show the activation patterns in S1 overlayed on magnified T1-weighted images for each finger. The location of the peak voxel in area 3b is indicated by blue crosshairs.

**Figure 3:**
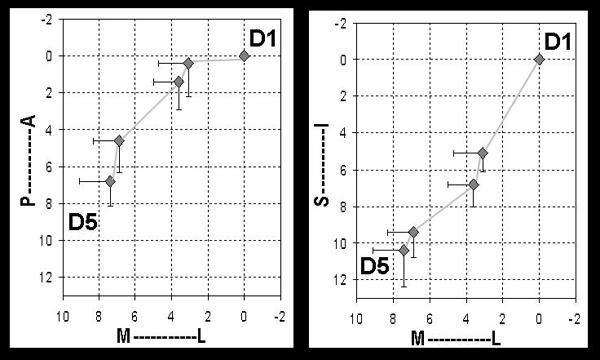
Distance to the thumb (D1) for each finger (mm), mean (diamonds) and SEM (errorbars), as presented in Table. The coordinates for D1 are defined as origo [0, 0, 0]. Distances to D1 are shown in the medial-lateral (M-L) direction, in the left panel also in the posterior-anterior (P-A) direction and in the right panel also in the superior-inferior (S-I) direction. The fingers are sequentially positioned more medially, posteriorly and superiorly.

We found activation in the anterior wall of the postcentral gyrus, defined as area 3b according to our operational definition during tactile stimulation (Fig 1). More pronounced activation was noticed frequently in the crown or posterior wall of the postcentral gyrus, defined as areas 1 and 2 (Fig 2). Similar observations have been made in other studies using both fMRI and PET [5,14]. According to studies in non-human primates the representation of the distal fingertips in area 1 points posteriorly, a finding confirmed in a recent fMRI-study on humans [15]. Area 2 then is the mirror-image of area 1 with the fingertips pointing anteriorly. The activation in the posterior wall might represent activation in both areas 1 and 2 localised at their meeting point, i.e. the fingertips. The larger cluster size of this activation is explained by the clusters arising from area 1 and 2 being contiguous and therefore additive. In the present study the activation of area 1 and 2 is probably due to hierarchical processing in the rostrocaudal direction within S1. A previous observation that electrical stimulation of the cutaneous afferents of the median nerve resulted in evoked potentials in area 3b after 30 msec while a potential in area 1 was seen after another 5 msec lends support to this assumption [16].

## Conclusion

In the group analysis, a somatotopic organisation for all the fingers in the hand representation of area 3b could be demonstrated using fMRI; the Euclidian distance between the thumb and the little finger was well comparable to that determined in previous studies. On the subject level the cortical somatosensory representation of the thumb was located laterally, anteriorly, and inferiorly to that of the little finger in 14 out of 17 subjects. The spatial localisation of the remaining fingers showed a less stringent somatotopic order when compared individually.

## Methods

### Subjects

Twenty healthy, self-reportedlly right-handed volunteers (6 male and 14 female, age 21–43 years, mean 29.4 years) were included in the study. The protocol was approved by the local ethics committee and written informed consent was obtained. All volunteers had normal images on a fluid-attenuated inversion recovery (FLAIR) sequence.

### Stimulation

Tactile stimulation consisted of brushing the glabrous skin of the two distal phalanges of each finger continuously forwards and backwards with a commercially available tooth brush. During the experiment, volunteers were positioned on the MR table with their right arm from the elbow down in a padded cast, that also provided support for the dorsal part of the hand. They were instructed to rest their arm against the magnet bore so that both arm and hand were relaxed. Pieces of soft cloth were placed between the fingers in order to avoid that stimulation also involved a neighbouring finger.

The frequency was 1 Hz; no forced pressure was exerted. Consistency was tested on a finger model firmly taped onto a computerized electronic scale (Biopac Systems, DA 100B, MP 100A; Macintosh Powerbook G3 with software AcqKnowledge 3.5): the intra-examiner error was 18% based on a mean pressure of 6.64 g, standard deviation 1.199 g.

### Imaging

MRI was performed using a 3 T head scanner (Siemens Allegra) with a quadrature birdcage coil. Morphological T1-weighted images with a resolution of 1 × 1 × 1 mm^3 ^using a magnetisation prepared gradient echo sequence (MPRAGE) were acquired. Functional echo-planar image volumes of the whole brain (number of slices = 49, thickness = 3 mm, voxel size = 3 × 3 × 3 mm^3^) sensitised to the Blood Oxygenation Level Dependent (BOLD)-effect (echo time = 30 ms) were acquired. Five scanning sessions were performed. Each session included 92 functional volumes with a temporal resolution of 3 seconds. The first two volumes in each session were discarded from further analysis to allow for initial T1-equilibrium effects.

### Experimental protocol

Fingers 1 to 5 (D1 = thumb, D5 = little finger) of the right hand were stimulated sequentially in separate sessions according to a block design that included four periods of stimulation and five of rest for each finger. The epoch length for both stimulation and rest periods was 30 seconds.

### Postprocessing

Image processing and analysis were carried out using the SPM99 soft ware package [17]. All functional images were resliced to a voxel size of 1.5 × 1.5 × 1.5 mm^3 ^and then realigned to the first image and coregistered to the T1-weighted image volume. All data were spatially filtered using an isotropic 4 mm, full-width, half-maximum Gaussian kernel. A high pass filter (cut off frequency 0.008 Hz) was applied to eliminate low frequency signal fluctuations. In order to preserve each subject's somatotopic arrangement in area 3b no normalization to a common brain atlas was performed.

### Data analysis

Functional data from two subjects were excluded due to major motion artefacts (subject no. 7) and global lack of activation (subject no.18). Task specific effects were estimated using the general linear model (GLM) that included a box car function convolved with the canonical hemodynamic response function in SPM99. The effect of sensory stimulation of each finger versus rest was determined using a one-sample t-test of pertinent linear contrasts of parameter estimates in each subject with a significance level of p < 0.001 (uncorrected). Then the spatial coordinates of the peak activation voxel in area 3b were determined. Due to lack of neuroanatomical landmarks, exact delineation of the cytoarchitectonically defined areas within S1 cannot be achieved in MR images. Therefore we used an operational definition based on cytoarchitectonic studies of S1 on 10 post-mortem brains. In >50 % of these brains area 3a was located in the fundus of the central sulcus, area 3b in the rostral bank of the postcentral gyrus and area 1 on its crown reaching down into the postcentral sulcus [18,19]. Fig 1 illustrates these locations, that continue along the central sulcus. As interareal borders vary across brains, the same authors constructed probability maps for each area by superimposing histological volumes of the individual brains on a computerized reference brain. Volumes of interest (VOIs) were defined for each area in which >50 % of the brains had a representation of that area. Despite close relationship of areas 3a, 3b and 1 in the postcentral gyrus, the three VOIs overlapped by <1% of their volumes. These probability maps were at hand when the spatial coordinates of the peak activation voxel in area 3b were determined. Activated peak voxels were labelled as belonging to area 3b when they were located within the anterior wall of the postcentral gyrus (Figure 1).

The spatial coordinates of the peak voxel for the thumb (D1) was defined as being at origo [0, 0, 0] in a 3D Cartesian coordinate system.When the spatial coordinates for all fingers were known in all subjects, somatotopy was assessed by determining the average distances for the whole group from each finger to the thumb.

Euclidian distances between fingers as well as from each finger to the thumb (D1) were calculated as: 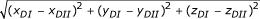


with x_DI_, y_DI _and z_DI _representing the coordinates of the finger DI and x_DII_, y_DII _and z_DII _representing the coordinates of DII in the three directions x, y, z in a system where the coordinates of D1 are at origo [0, 0, 0].

### Statistics

Each finger's distance to the first finger was compared to that of the directly neighbouring fingers using the Wilcoxon matched pairs test with a significance level of p < 0.05. The Euclidian distances to the first finger were compared for each finger using the Wilcoxon matched pairs test with a significance level of p < 0.05.

## Authors' contributions

DvW conceived of the study, performed the image analysis and drafted the manuscript. PF guided the image analysis. JO designed the MR-parameters. EML participated in the design of the study. BR and GL initiated and conducted the stimulation consistency study. All authors read and approved the final manuscript.

## Figures and Tables

**Table:** 

	**Distance to finger 1**			
**Finger**	***x L → M***	***y A → P***	***z I → S***	***Euclidian***
**D1**	0 (0)	0 (0)	0 (0)	0 (0)
**D2**	2.9 (1.6)	0.4 (1.8)	4.8 (1.0)	10.6 (1.5)
**D3**	3.6 (1.4)	1.4 (1.5)	6.8 (1.2)	11.2 (1.5)
**D4**	6.9 (1.4)	4.6 (1.7)	9.4 (1.4)	15.4 (1.7)
**D5**	7.4 (1.7)	6.8 (1.2)	10.4 (2)	17.2 (2.0)

Distance from each finger (D2, D3, D4, D5) to the thumb (D1) ± standard error of the mean (SEM) (mm). The lateral-to-medial direction is named 'x', the anterior-to-posterior direction 'y' and the inferior-to-superior direction 'z'. Underlined are those distances to D1 that differ significantly from the distance of the previous finger to D1 (Wilcoxon matched pairs test, p < 0.05).
